# Expanding Protection Motivation Theory to Explain Willingness of COVID-19 Vaccination Uptake among Taiwanese University Students

**DOI:** 10.3390/vaccines9091046

**Published:** 2021-09-19

**Authors:** Po-Ching Huang, Ching-Hsia Hung, Yi-Jie Kuo, Yu-Pin Chen, Daniel Kwasi Ahorsu, Cheng-Fang Yen, Chung-Ying Lin, Mark D. Griffiths, Amir H. Pakpour

**Affiliations:** 1 Institute of Allied Health Sciences, College of Medicine, National Cheng Kung University, Tainan 701401, Taiwan; hh780705@hotmail.com (P.-C.H.); chhung@mail.ncku.edu.tw (C.-H.H.); 2 Department of Physical Therapy, College of Medicine, National Cheng Kung University, Tainan 701401, Taiwan; 3 Department of Orthopedic Surgery, Wan Fang Hospital, Taipei Medical University, Taipei 116081, Taiwan; benkuo5@tmu.edu.tw (Y.-J.K.); 99231@w.tmu.edu.tw (Y.-P.C.); 4 Department of Orthopedic Surgery, School of Medicine, College of Medicine, Taipei Medical University, Taipei 106339, Taiwan; 5 Department of Rehabilitation Sciences, The Hong Kong Polytechnic University, Hung Hom, Hong Kong, China; daniel.ahorsu@connect.polyu.hk; 6 Department of Psychiatry, School of Medicine College of Medicine, Kaohsiung Medical University, Kaohsiung 807377, Taiwan; 7 Department of Psychiatry, Kaohsiung Medical University Hospital, Kaohsiung 807377, Taiwan; 8 College of Professional Studies, National Pingtung University of Science and Technology, Pingtung 91201, Taiwan; 9 Department of Occupational Therapy, College of Medicine, National Cheng Kung University, Tainan 701401, Taiwan; 10 Department of Public Health, National Cheng Kung University Hospital, College of Medicine, National Cheng Kung University, Tainan 701401, Taiwan; 11 International Gaming Research Unit, Psychology Department, Nottingham Trent University, Nottingham NG1 4FQ, UK; mark.griffiths@ntu.ac.uk; 12 Social Determinants of Health Research Center, Research Institute for Prevention of Non-Communicable Diseases, Qazvin University of Medical Sciences, Qazvin 34197-59811, Iran; apakpour@qums.ac.ir; 13 Department of Nursing, School of Health and Welfare, Jönköping University, SE-551 11 Jönköping, Sweden

**Keywords:** coping appraisal, COVID-19 vaccination, intention, perceived knowledge, protection motivation theory, vaccine hesitancy

## Abstract

Vaccination appears to be one of the effective strategies to control the COVID-19 pandemic. However, the challenge of vaccine hesitancy may lower the uptake rate and affect overall vaccine efficacy. Being a low-risk group in terms of serious consequences of infection, university students may possess low motivation to get vaccinated. Therefore, an expanded Protection Motivation Theory (PMT) incorporating perceived knowledge, adaptive response, and maladaptive response was proposed to investigate the COVID-19 vaccination intention among Taiwanese university students. University students (*n* = 924; 575 males; mean age = 25.29 years) completed an online survey during January to February 2021. The proposed expanded PMT model was examined using structural equation modeling (SEM). The results showed that perceived knowledge was significantly associated with coping appraisal (standardized coefficient (β) = 0.820; *p* < 0.001), and coping appraisal was significantly associated with adaptive response (β = 0.852; *p* < 0.001), maladaptive response (β = 0.300; *p* < 0.001) and intention (β = 0.533; *p* = 0.009). Moreover, maladaptive response (β = −0.173; *p* = 0.001) but not adaptive response (β = 0.148; *p* = 0.482) was significantly and negatively associated with intention. The present study’s results demonstrated a positive path between perceived knowledge, coping appraisal, and intention among university students. Therefore, improving knowledge among this population may increase the intention to uptake the vaccine.

## 1. Introduction

A new coronavirus, severe acute respiratory syndrome coronavirus 2 (SARS-CoV-2) emerged in 2019 and was rapidly transmitted worldwide. Transmission of respiratory secretion from a carrier spreads SARS-CoV2 by infecting the respiratory system [1,2], causing varied respiratory symptoms from mild upper respiratory tract infection to the critical illness of fatal pneumonia [3]. Due to its high contagiousness and mortality rate, the World Health Organization (WHO) had declared it a health-threatening pandemic and named the disease of SARS-CoV-2 infection as coronavirus disease 2019 (COVID-19) [4]. At the time of writing (9 August 2021), more than four million individuals have died [5] and the numbers continue to rise, especially due to faster spreading COVID-19 mutations [6]. Apart from preventive policies to inhibit the spread of the virus (e.g., border controls, city lockdowns, spatial distancing, quarantining, etc.) launched by many governments worldwide, vaccination appears to be one of the most effective strategies to defeat the pandemic [7]. Fortunately, the development of vaccines has been a significant breakthrough [8] and more than three billion doses of vaccines have been administered to date [5].

However, vaccination uptake has been a challenging issue for almost all countries worldwide, because at least a suggested 70% to 80% of the vaccinated population is recommended to best control the pandemic considering the emergence of more contagious variants [9]. Unfortunately, some individuals are still hesitant about receiving a vaccine despite the gradually increasing proportion of populations being vaccinated [10,11,12]. Several reasons for vaccine hesitancy have been reported [11,12,13,14]. These reasons include population characteristics, previous vaccination history, general vaccination beliefs, and beliefs and attitudes toward the pandemic [15,16]. The intention to initially have the vaccine is the key element that facilitates individuals to get vaccinated [17]. Therefore, it is crucial and important for both healthcare providers and policymakers to identify the factors associated with vaccination uptake intention.

In order to examine individuals’ intention to get a COVID-19 vaccination, a well-established theory that can be applied in the current circumstances is of great benefit. Protection Motivation Theory (PMT), a type of social cognition theory, is a good candidate theory to understand the underlying reasons for individuals’ intention to get a COVID-19 vaccination. The PMT predicts the self-protective motivation of individuals towards a perceived threat [18,19]. More specifically, threat appraisal (strategy to evaluate the severity of an event) and coping appraisal (strategy to generate coping behavior) are key elements in PMT that shape an individual’s motivation/intention to perform protective behavior [20]. Appraisal of threat is derived from individuals’ beliefs concerning the risk to their health status, while the appraisal of coping is derived from the potential strategies that individuals can engage in and their possible effect [21]. When a health threat event appears, an estimated threat appraisal would be generated by evaluating the severity and vulnerability in view of the individual’s perceived knowledge. A coping appraisal based on previous experiences would also be generated in the system by evaluating self-efficacy and response efficacy. As a result, a determined protective motivation would be engendered through the mutual interaction of threat appraisal and coping appraisal, further leading to the enacting of a protective behavior [22].

Because the appraisal may be dependent on individuals’ perceived knowledge, such knowledge should be an important factor when explaining the willingness of COVID-19 vaccination uptake, although it is not specifically mentioned in the PMT. Perceived knowledge indicates the perceived information regarding the specific event. Previous studies had focused on its contribution to intention formation. One study reported an abundant knowledge background among university students toward COVID-19, others have demonstrated that knowledge significantly influences adherence to social restriction policies against COVID-19 in the investigated populations [23,24].

Apart from perceived knowledge, when an individual reacts to an environmental stimulus, a protective or precautionary behavior might be performed, and the ability to take appropriate action is deemed to be an adaptive response. On the other hand, the ability to make a decision that is against the individual’s own interest is deemed a maladaptive response [25,26]. The process of coping appraisal may lead to either adaptive or maladaptive responses [27]. Studies have suggested that without proper coping information, threat information and fear may directly lead the individuals to engage in maladaptive responses, further enhancing the unwanted behavior [28]. In contrast, accurate information may strengthen the adaptive (rather than the maladaptive) response [29], facilitating individuals to take protective health behavior.

In the present study, university students were targeted because they are a low-risk group in terms of serious consequences of infection or only have asymptomatic symptoms if infected. Therefore, this may result in low motivation to get vaccinated. The low serious infection rate and lack of priority in vaccination uptake may explain the poor intention to get vaccinated against COVID-19 among university students [30,31]. Nevertheless, the rapid development of COVID-19 vaccines together with full worldwide coverage is essential to effectively control the pandemic [32]. In other words, all countries need to have sufficient vaccination coverage to build up a global protective network from COVID-19 infection. Therefore, it is important to identify intention formation among this population in order to initiate beneficial vaccination behavior.

**Figure 1:**
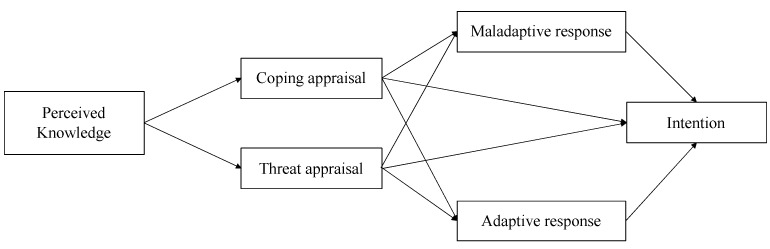
Proposed model utilizing protection motivation theory (PMT) to explain the intention to get COVID-19 vaccinated.

In order to investigate the involvement of possible factors related to the formation of vaccination intention, PMT was used as the main framework alongside other important factors (perceived knowledge, adaptive response, and maladaptive response) to test the proposed model. According to PMT and literature concerning perceived knowledge, the present study hypothesized that (1) perceived knowledge would have a positive association with coping appraisal; (2) coping appraisal would directly contribute to intention; (3) coping appraisal would also contribute to both adaptive and maladaptive response; and (4) both adaptive and maladaptive responses would have associations with intention. All hypotheses are illustrated in Figure 1.

## 2. Materials and Methods

### 2.1. Participants and Procedure

*Google Forms* was used to create an online survey for data collection. Two inclusion criteria of (i) being aged 20 years or above and (ii) currently studying at a university, were used to define participant eligibility. The research team asked the departments and faculties of Taiwan universities to advertise the survey to enroll potential participants. The survey took place between 5 January and 5 February 2021—a period when the Taiwan government still had no vaccines (Taiwan had the first batch of vaccines available on 3 March 2021). The study protocol was approved by the Institutional Review Board (IRB) of the Kaohsiung Medical University Chung-Ho Memorial Hospital (IRB ref: KMUHIRB-EXEMPT(I)-20200019). An e-consent form was provided in the online survey. Several ways were used to ensure that the respondents were university students: (i) on the first page of the survey, we specifically asked whether the participant was a university student. If the participant answered ‘no’, the survey shut down directly and the participant was unable continue the survey; (ii) the participants were asked to indicate which major they are currently studying; and (iii) the online survey was distributed via university faculties to university students. A total of 932 surveys were begun but eight were excluded due to inadequate responses (i.e., repeated responses or unrealistic personal information such as reporting age as being 100 years). Therefore, a total of 924 responses were used for analysis. There were no missing data as the online survey could only be submitted if all the survey items were completed.

### 2.2. Measures

The demographics collected in the present survey included the participants’ gender (male or female), age (in years), educational level (undergraduate or postgraduate), major subject of study (medicine, nursing, pharmacology, social work, occupational or physical therapy, psychology, speech therapy, medical science and biotechnology, engineering, science, psychosocial science, art and design, electrical engineering and computer science, liberal arts, others), and the marital status (i.e., single, married, other). In addition, detailed item descriptions of the measures for the following constructs are provided in Appendix A.

*Perceived knowledge* was defined as perceived knowledge about the COVID-19 vaccine and assessed using three items rated on a seven-point Likert scale (1 = strongly disagree; 7 = strongly agree). A higher score on each item indicates a higher knowledge level regarding the COVID-19 vaccine. The three-item perceived knowledge scale in the present study had very good internal consistency (α = 0.845).

*Coping appraisal* was defined as strategies to cope with vaccine injection and assessed using five items rated on a seven-point Likert scale (1 = strongly disagree; 7 = strongly agree). A higher score on each item indicates a higher agreement of receiving a vaccine injection as a strategy to protect themselves from the pandemic. The five-item coping appraisal scale in the present study had very good internal consistency (α = 0.836).

*Threat appraisal* was defined as strategy to evaluate the severity of COVID-19 pandemic and was assessed using five items. Two of five items are rated on a five-point Likert scale (1 = not at all concerned; 5 = extremely concerned). A higher score on each item indicates less concern of being infected. Two of five items are rated on a seven-point Likert scale (1 = very probable; 7 = not probable). A higher score on each item indicates a lesser chance of being infected. One of the five items is rated on a ten-point visual analogue scale (1 = strongly disagree; 10 = strongly agree). A higher score on the item indicates a lesser concern of being infected. The five-item threat appraisal scale in the present study had adequate internal consistency (α = 0.689).

*Maladaptive response* was defined as negative thoughts of vaccine injection and assessed using one item rated on a seven-point Likert scale (1 = strongly disagree; 7 = strongly agree). A higher score on the item indicates a higher level regarding the negative thoughts toward vaccine injection.

*Adaptive response* was defined as positive thoughts of vaccine injection and assessed using one item rated on a seven-point Likert scale (1 = strongly disagree; 7 = strongly agree). A higher score on the item indicates a higher agreement of considering vaccination as a wellbeing improvement.

*Intention* was defined as intention to receive the vaccine injection and assessed using one item rated on a ten-point visual analogue scale (1 = strongly disagree; 10 = strongly agree). A higher score on the item indicates a higher willingness to receive vaccination afterwards.

### 2.3. Data Analysis

A chi-square test was used to exam the differences in sex and study major distributions between the whole Taiwanese university student population [33,34] and the present study’s sample. Means and standard deviations of descriptive statistics were calculated to understand the characteristic of the participants, including their demographics and scores of each factor in the proposed model. The participants were divided into two subgroups of students (i.e., students majoring in medicine-related programs and those majoring in non-medicine-related programs) to examine whether any studied variable in the present study was significantly different between the two subgroups. Independent *t*-tests were used for continuous variables and chi-square tests for categorical variables. Pearson correlation coefficients were used to exam the bivariate associations between each factor listed in the proposed model and *type 1 error* was adjusted to 0.0033 (i.e., 0.05/15) indicating an appropriate significance level according to Bonferroni correction. Structural equation modeling (SEM) with the estimator of diagonally weighted least squares was set to examine if the collected data fit with the proposed model (Figure 1) for the entire sample and the two subgroups (i.e., students majoring in medicine-related programs and those majoring in non-medicine-related programs). Four indices were used to evaluate if the proposed model was supported. The indices included comparative fit index (CFI), Tucker–Lewis index (TLI), root mean square error of approximation (RMSEA), and standardized root mean squared residual (SRMR) [35]. The level of CFI and TLI should be >0.95 and the RMSEA and SRMR should be <0.08, respectively. When the fit indices are satisfactory, the path coefficients in the SEM are further scrutinized. The SEM was performed using the lavaan package in the R software [36] and the remaining data analyses were carried out using the SPSS 17.0 [37].

## 3. Results

Table 1 shows that the present study’s sample as compared with the entire Taiwanese university student population had significantly more male students and students majoring in medicine. Participants were mainly male (*n* = 575, 62.2%) and single (*n* = 867, 93.8%). The participants’ mean age was 25.29 years (SD = 6.30). Table 2 provides the mean scores of the studied variables in the present study. Regarding the features between students majoring in medicine and those not majoring in medicine, there were significantly more female students and fewer married individuals among those majoring in medicine compared with those not majoring in medicine. The students majoring in medicine also reported lower levels of adaptive response and higher level of threat appraisal than those not majoring in medicine (Table 2). Moreover, the bivariate associations between the studied variables are provided in Table 3. More specifically, significantly moderate associations were identified between perceived knowledge, adaptive response, coping appraisal, and intention (*r* = 0.477 to 0.618; all *p*-values < 0.001). Additionally, perceived knowledge was significantly and strongly associated with adaptive response (*r* = 0.716; *p* < 0.001). Furthermore, coping appraisal was significantly and strongly associated with perceived knowledge and adaptive response (*r* = 0.794 to 0.823; all *p*-values < 0.001).

**Figure 2:**
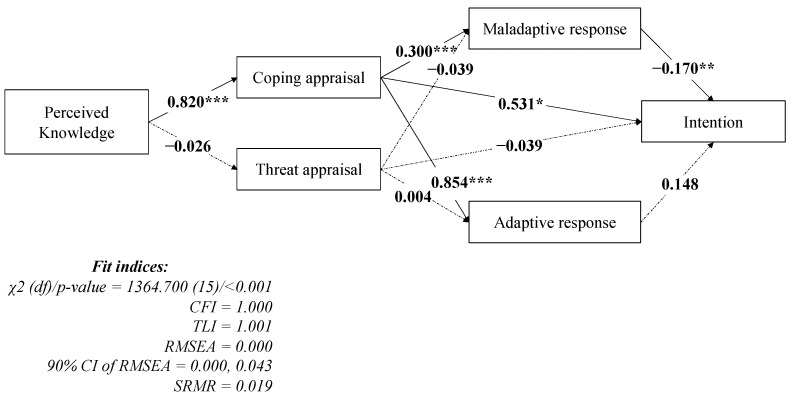
Confirmed model explaining the intention to get COVID-19 vaccination. Coefficients are presented using standardized coefficients. Solid lines indicate significant pathways while dashed lines indicate non-significant pathways. * *p* < 0.05; ** *p* < 0.01; *** *p* < 0.001. CFI = Comparative fit index; TLI = Tucker–Lewis index; RMSEA = root mean square error of approximation; SRMR = standardized root mean square residual.

**Figure 3:**
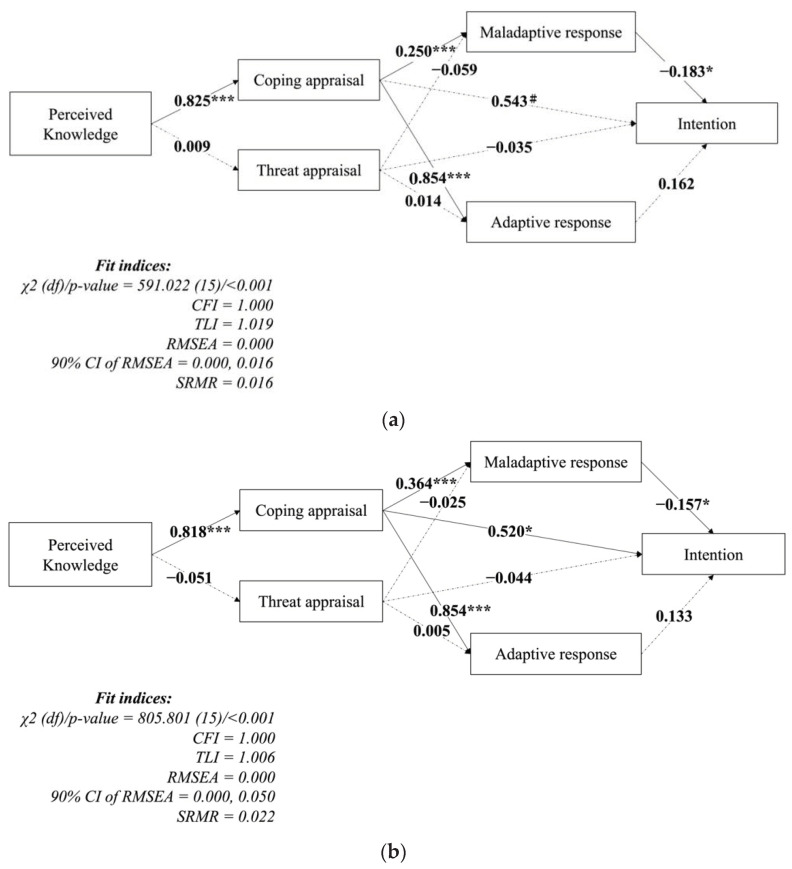
Confirmed model explaining the intention to get COVID-19 vaccination of (**a**) students majoring in medicine-related programs and (**b**) students majoring in non-medicine-related programs. Coefficients are presented using standardized coefficients. Solid lines indicate significant pathways while dashed lines indicate non-significant pathways. * *p* < 0.05; *** *p* < 0.001; ^#^ *p* < 0.01. CFI = Comparative fit index; TLI = Tucker–Lewis index; RMSEA = root mean square error of approximation; SRMR = standardized root mean square residual.

The SEM model demonstrated a well-fitted model (Figure 2), as supported by all of the fit indices (CFI = 1.000; TLI = 1.001; RMSEA = 0.000; and SRMR = 0.019), except for the significant χ^2^ test (*p* < 0.001). The SEM model further showed that perceived knowledge (standardized coefficient (β) = 0.820; *p* < 0.001) was significantly associated with coping appraisal. Coping appraisal was significantly associated with intention (β = 0.531; *p* = 0.010), maladaptive response (β = 0.300; *p* < 0.001), and adaptive response (β = 0.854; *p* < 0.001). Maladaptive response (β = −0.170; *p* = 0.001) but not adaptive response (β = 0.148; *p* = 0.703) was significantly negatively associated with intention. In addition, threat appraisal showed no significant correlation with any item in the proposed model. The SEM model fitted well with the subgroups’ data (Figure 3), as supported by all the fit indices, except for the significant χ^2^ test (*p* < 0.001). In addition, the path coefficients in the SEM of subgroup who majored in medicine showed a marginally significant correlation, whereas the subgroup who majored in non-medicine was similar to those in the entire sample SEM. This indicates that students majoring in medicine and those not majoring in medicine shared similar psychological mechanisms for their intention regarding COVID-19 vaccination uptake.

## 4. Discussion

The present study showed the potential mechanism (i.e., expanded PMT) explaining the intention for COVID-19 vaccination uptake among university students in Taiwan, a region with relatively low risk of COVID-19 infection [38,39,40]. More specifically, the expanded PMT tested in the present study indicated that perceived knowledge was significantly related to coping appraisal, and further with intention of COVID-19 vaccination uptake. The positive association between coping appraisal and intention found in the present study concurs with prior findings on PMT [15,41]. This significant association further indicates that the effect of coping appraisal on intention formation of COVID-19 vaccination uptake could be applied during health-threat pandemics, such as that with COVID-19 [42,43,44,45]. Additionally, most studies have demonstrated that self-efficacy is an effective factor in intention formation and behavior engagement [45,46]. Therefore, the use of PMT appears to be promising in shaping university students’ intention to get a COVID-19 vaccination, and subsequently, increase the coverage of COVID-19 vaccination across the country.

Apart from PMT, perceived knowledge was found to be strongly associated with coping appraisal. In other words, the amount of perceived knowledge may affect the adoption of coping strategies among university students, and this finding agrees with a previous study [23]. Moreover, knowledge has been evidenced as one of the key elements in the control of pandemics [42] because individuals with high levels of knowledge are more likely to generate protection intentions [23]. Knowledge may also improve the engagement of precautionary behavior and increase the self-efficacy of coping appraisal [41]. In addition, a sense of fear might prompt the individuals to search for information regarding the COVID-19 vaccine, further enhancing the knowledge or perceived knowledge of their own. In other words, the improvement of the perceived knowledge could also be facilitated by fear. Therefore, adding perceived knowledge in the original PMT appears appropriate and can increase the efficacy of the PMT to improve university students’ intention of COVID-19 vaccination uptake.

The present study found that coping appraisal was strongly associated with adaptive responses and moderately negatively associated with maladaptive responses. A stressor (e.g., the COVID-19 pandemic) may induce an individual’s responses via coping appraisal and such responses can be positive (i.e., adaptive responses) or negative (i.e., maladaptive responses) [29,47]. If a sense of losing control is perceived by individuals, they may start feeling depressed and anxious. In order to regain control, individuals might adopt maladaptive responses [48]. The most common strategy of maladaptive response is avoidance, which would further affect the intention of adopting protective behavior [49]. Because the question related to maladaptive response in the present study was to investigate the perceived pressure of COVID-19 vaccination, it anticipates a low association between maladaptive response and intention, although the association was significant. As for adaptive response, previous research had suggested that coping appraisal could enhance both adaptive response [27] and intention [45]. In other words, coping appraisal might have a positive effect on protective behavior which could affect both the adaptive response and intention of individuals. However, a non-significant association between adaptive response and intention was found in the present study. A rational assumption is that the effect of adaptive response to enhance intention might be diminished due to the direct interaction between coping appraisal and intention. Consequently, adaptive response was unable to demonstrate a significant association with intention.

The present study has several limitations. First, the participants’ data were collected using a convenience sampling method. More specifically, the present authors distributed an online survey with the assistance of university departments, faculties, and colleagues. Therefore, the collected data could have a similar pattern due to the regional effect because the survey distribution might be restricted within the participants sharing similar contexts and features. The representativeness of the present study is therefore limited. Second, the study adopted a cross-sectional design, which can be criticized for its low evidence in cause-and-effect relationships due to the lack of a temporal element in data collection. More specifically, the data collection time of the latent variables were not in an ordered sequence, which indicates that the proposed model is possible to have a different order among these latent variables. For example, “threat appraisal” of SARS-CoV-2 risk could proceed instead of following “perceived knowledge” of vaccines at the time of the survey (i.e., before the availability of vaccines). Third, the collected data were all self-reports utilizing perceived evaluation. This means there could have been some biases and misrepresentations. For example, the single-rater bias (aka common method bias) and the social desirability bias (e.g., the participants in the present study might have pretended to have a high motivation to get COVID-19 vaccinated). Fourth, although several methods were used to ensure the participants were students, it is possible that some participants were not students and obtained access to the online survey via sources unknown to the research team. There may also be some differences between respondents and non-respondents. More specifically, respondents in the present study may pay more attention or care more about COVID-19 than the non-respondents. Therefore, the generalizability of the present study’s results cannot be applied to non-responders. Fifth, quality control items were not included in the present study (e.g., using a simple calculation after item verification to ensure the participants were focused on the survey questions). Therefore, future replication studies should consider the use of quality control items. Sixth, the estimated eligible participants were about 20,000. Therefore, present study reached approximately 4.7% of eligible participants, which is a relatively low participation rate. Seventh, the present study’s sample (as compared with the entire Taiwan university student population) had more male students and more students majoring in medicine. Nevertheless, the present results still provided a clear path regarding the formation of the intention to get COVID-19 vaccination among a sample of Taiwanese university students. The present study’s findings may further provide government health policymakers with some directions and insights to improve the COVID-19 vaccination uptake and subsequently fulfill vaccination target rates.

## 5. Conclusions

The present study used PMT as the main theoretical framework with the incorporation of other relevant factors (i.e., perceived knowledge, adaptive response, and maladaptive response), to illustrate that expanding PMT was effective in investigating COVID-19 vaccination intention among university students in Taiwan. The results showed a clear path of the association between perceived knowledge and coping appraisal, further associated with intention. Based on the study’s results, the government’s health department could provide knowledge regarding COVID-19 vaccination to improve individual’s perceived knowledge. Individuals could adopt anxiety reduction methods such as mindfulness during the lockdown to facilitate self-control and prompt the coping appraisal with regard to vaccination confidence. Either way may benefit individuals in reducing vaccine hesitancy and enhance the vaccination uptake rate. It is also recommended that future studies focus on exploring the relative factors that may affect intention formation among other population groups to increase the vaccination rate.

## Figures and Tables

**Table 1:** Comparing sex and study major between the present study’s sample and the entire Taiwanese university student population.

Variables	N (%)		χ^2^	*p*-Value
	Entire Taiwanese University Students (*n* = 1,203,429)	Respondents in the Present Study (*n* = 924)		
Sex			60.54	<0.001
Male	594,816 (49.4)	575 (62.2)		
Female	608,613 (50.6)	349 (37.8)		
Study major			1230.94	<0.001
Major in medicine	114,330 (9.5)	401 (43.4)		
Major in non-medicine	1,089,099 (90.5)	523 (56.6)		

**Table 2:** Characteristics of participants (*n* = 924).

			Medicine Major (*n* = 401)	Non-Medicine Major (*n* = 523)	
	Mean (SD) or N (%)	Possible Range	Mean (SD) or N (%)	Mean (SD) or N (%)	*p*-Value
Sex (male)	575 (62.2)		273 (68.1)	302 (57.7)	0.001
Age	25.3 (6.3)		25.3 (5.5)	25.3 (6.9)	0.956
Marital status					0.001
Married	49 (5.3)		366 (91.3)	502 (96.0)	
Single	867 (93.8)		28 (7.0)	21(4.0)	
Others	7 (0.08)		7 (1.8)	0 (0)	
^a^ Perceived knowledge	5.08 (1.23)	1–7	5.05 (1.26)	5.10 (1.20)	0.498
^b^ Coping appraisal	4.80 (0.90)	1–7	4.75 (0.92)	4.83 (0.89)	0.191
^c^ Threat appraisal	4.18 (0.92)	1–6.8	4.28 (0.90)	4.10 (0.93)	0.002
^d^ Maladaptive response	4.86 (1.42)	1–7	4.88 (1.45)	4.85 (1.40)	0.771
^e^ Adaptive response	5.22 (1.29)	1–7	5.10 (1.32)	5.31 (1.26)	0.014
^f^ Intention	6.59 (2.21)	1–10	6.49 (2.29)	6.67 (2.15)	0.219
Score 1	25 (2.71)		15 (3.74)	10 (1.91)	
Score 2	26 (2.81)		15 (3.74)	11 (2.10)	
Score 3	46 (4.98)		23 (5.74)	23 (4.40)	
Score 4	55 (5.95)		19 (4.74)	36 (6.88)	
Score 5	111 (12.01)		41 (10.22)	70 (13.38)	
Score 6	142 (15.37)		64 (15.96)	78 (14.91)	
Score 7	164 (17.75)		72 (17.96)	92 (17.59)	
Score 8	176 (19.05)		79 (19.70)	97 (18.55)	
Score 9	98 (10.61)		43 (10.72)	55 (10.52)	
Score 10	81 (8.77)		30 (7.48)	51 (9.75)	

^a^ Perceived knowledge: perceived knowledge about the COVID-19 vaccine; ^b^ Coping appraisal: strategies to cope with vaccine injection; ^c^ Threat appraisal: strategy to evaluate the severity of COVID-19 pandemic; ^d^ Maladaptive response: negative thought of vaccine injection; ^e^ Adaptive response: positive thought of vaccine injection; ^f^ Intention: intention to receive the vaccine injection.

**Table 3:** Correlations between study variables.

Variable	1	2	3	4	5	6
**1 Perceived knowledge**						
*r*	1					
*p*-value	-					
**2 Coping appraisal**						
*r*	0.794	1				
*p*-value	<0.001 *	-				
**3 Threat appraisal**						
*r*	−0.017	−0.042	1			
*p*-value	0.604	0.206	-			
**4 Maladaptive response**						
*r*	0.293	0.246	−0.046	1		
*p*-value	<0.001 *	<0.001 *	0.165	-		
**5 Adaptive response**						
*r*	0.716	0.823	−0.017	0.272	1	
*p*-value	<0.001 *	<0.001 *	0.616	<0.001 *	-	
**6 Intention**						
*r*	0.477	0.618	−0.047	0.029	0.558	1
*p*-value	<0.001 *	<0.001 *	0.152	0.374	<0.001 *	-

* Indicates significance using Bonferroni correction, which adjusted the Pearson’s *r* to 0.0033.

## Data Availability

The data will be available upon reasonable request to the corresponding authors.

## Appendix A. The Latent Variables with Corresponding Items, Likert-Scale, and Cronbach Coefficients

**Perceived knowledge (α = 0.845)**

1. I know exactly how the vaccine will protect me from getting the COVID-19
  我非常清楚疫苗將會如何保護我免於感染新冠肺炎。
  1	2	3	4	5	6	7
  Strongly disagree	Disagree	Somewhat disagree	Neither agree or disagree	Somewhat agree	Agree	Strongly agree
  完全不同意	很不同意	有一點不同意	沒同意也沒不同意	有一點同意	很同意	完全同意
2. I know exactly by what kind of mechanism the vaccine will activate to protect my body fight against the COVID-19 virus
  我了解疫苗將經由何種機制來幫助我的身體對抗新冠肺炎病毒。
  1	2	3	4	5	6	7
  Strongly disagree	Disagree	Somewhat disagree	Neither agree or disagree	Somewhat agree	Agree	Strongly agree
  完全不同意	很不同意	有一點不同意	沒同意也沒不同意	有一點同意	很同意	完全同意
3. The COVID-19 vaccine plays an important role in protecting our life
  新冠肺炎疫苗將會在保護我和其他人的生命上扮演重要角色。
  1	2	3	4	5	6	7
  Strongly disagree	Disagree	Somewhat disagree	Neither agree or disagree	Somewhat agree	Agree	Strongly agree
  完全不同意	很不同意	有一點不同意	沒同意也沒不同意	有一點同意	很同意	完全同意

**Coping appraisal (α = 0.836)**

4. Receiving a vaccine injection is going to reduce the possibility to get COVID-19
  注射疫苗將能非常有效地保護我免於感染新冠肺炎。
  1	2	3	4	5	6	7
  Strongly disagree	Disagree	Somewhat disagree	Neither agree or disagree	Somewhat agree	Agree	Strongly agree
  完全不同意	很不同意	有一點不同意	沒同意也沒不同意	有一點同意	很同意	完全同意
5. Receiving the COVID-19 vaccine is important to me
  對我來說，注射新冠肺炎疫苗將會是重要的事。
  1	2	3	4	5	6	7
  Strongly disagree	Disagree	Somewhat disagree	Neither agree or disagree	Somewhat agree	Agree	Strongly agree
  完全不同意	很不同意	有一點不同意	沒同意也沒不同意	有一點同意	很同意	完全同意
6. Receiving the vaccine will significantly reduce the possibility of getting COVID-19
  注射疫苗將能大幅降低我感染新冠肺炎的機會。
  1	2	3	4	5	6	7
  Strongly disagree	Disagree	Somewhat disagree	Neither agree or disagree	Somewhat agree	Agree	Strongly agree
  完全不同意	很不同意	有一點不同意	沒同意也沒不同意	有一點同意	很同意	完全同意
7. Receiving the COVID-19 vaccine will have a positive influence on my health status
  注射新冠肺炎疫苗將會對我的健康有正面的影響。
  1	2	3	4	5	6	7
  Strongly disagree	Disagree	Somewhat disagree	Neither agree or disagree	Somewhat agree	Agree	Strongly agree
  完全不同意	很不同意	有一點不同意	沒同意也沒不同意	有一點同意	很同意	完全同意
8. I will make the decision by myself in the future whether to receive the COVID-19 vaccine or not
  未來我將會幫自己決定是否要注射新冠肺炎疫苗。
  1	2	3	4	5	6	7
  Strongly disagree	Disagree	Somewhat disagree	Neither agree or disagree	Somewhat agree	Agree	Strongly agree
  完全不同意	很不同意	有一點不同意	沒同意也沒不同意	有一點同意	很同意	完全同意

**Threat appraisal (α = 0.689)**

9. If I get a flu-like symptom tomorrow, I will be very worried.
  如果您明天出現類似流行性感冒的症狀，您可能會：
  1	2	3	4	5	6	7
  Strongly disagree	Disagree	Somewhat disagree	Neither agree or disagree	Somewhat agree	Agree	Strongly agree
  完全不同意	很不同意	有一點不同意	沒同意也沒不同意	有一點同意	很同意	完全同意
10. In the past week, I was worried that I might get COVID-19
  在過去一星期，您是否曾經擔心自己會得到新冠肺炎?
  1	2	3	4	5	6	7
  Strongly disagree	Disagree	Somewhat disagree	Neither agree or disagree	Somewhat agree	Agree	Strongly agree
  完全不同意	很不同意	有一點不同意	沒同意也沒不同意	有一點同意	很同意	完全同意
11. Please rate your current worry level concerning the COVID-19 pandemic from 1 (not worried at all) to 10 (extremely worried)
  請從1到10之間選個數字，來代表您現在對於新冠肺炎的擔心程度高低。
12. In the following one month, I think it’s likely that I will get COVID-19
  您覺得自己未來一個月得到新冠肺炎的可能性有多高?
  1	2	3	4	5	6	7
  Strongly disagree	Disagree	Somewhat disagree	Neither agree or disagree	Somewhat agree	Agree	Strongly agree
  完全不同意	很不同意	有一點不同意	沒同意也沒不同意	有一點同意	很同意	完全同意
13. Compared to other people (except my family), I think it’s likely that I will get COVID-19
  和您家人以外的他人比較的話，您覺得自己未來一個月得到新冠肺炎的機會有多高?
  1	2	3	4	5	6	7
  Strongly disagree	Disagree	Somewhat disagree	Neither agree or disagree	Somewhat agree	Agree	Strongly agree
  完全不同意	很不同意	有一點不同意	沒同意也沒不同意	有一點同意	很同意	完全同意

**Adaptive response**

14. I think that receiving the vaccine will make a great contribution to my happiness and wellbeing in the future
  新冠肺炎疫苗將會對於我的健康和幸福有著重要貢獻。
  1	2	3	4	5	6	7
  Strongly disagree	Disagree	Somewhat disagree	Neither agree or disagree	Somewhat agree	Agree	Strongly agree
  完全不同意	很不同意	有一點不同意	沒同意也沒不同意	有一點同意	很同意	完全同意

**Maladaptive response**

15. I think it will be stressful for me to receive the vaccine in the future
  我預測未來我將會在注射新冠肺炎疫苗這件事情上感受到壓力。
  1	2	3	4	5	6	7
  Strongly disagree	Disagree	Somewhat disagree	Neither agree or disagree	Somewhat agree	Agree	Strongly agree
  完全不同意	很不同意	有一點不同意	沒同意也沒不同意	有一點同意	很同意	完全同意

**Intention**

16. Please rate your intention to get COVID-19 vaccination in the future from 1 (totally unwilling to) to 10 (extremely willing to)
  請從1到10之間選個數字，來代表您現在對於日後願意接受新冠肺炎疫苗注射的程度。

## References

[1] Thomas P., Baldwin C., Bissett B., Boden I., Gosselink R., Granger C.L., Hodgson C., Jones A.Y., Kho M.E., Moses R. (2020). Physiotherapy management for COVID-19 in the acute hospital setting: Clinical practice recommendations. J. Physiother..

[2] Carlos W.G., Cruz C.S.D., Cao B., Gross J.E., Pasnick S., Jamil S. (2020). COVID-19: How do we stay safe?. Am. J. Respir. Crit. Care Med..

[3] Felten-Barentsz K.M., van Oorsouw R., Klooster E., Koenders N., Driehuis F., Hulzebos E.H.J., van der Schaaf M., Hoogeboom T.J., van der Wees P.J. (2020). Recommendations for hospital-based physical therapists managing patients with COVID-19. Phys. Ther..

[4] Velavan T.P., Meyer C.G. (2020). The COVID-19 epidemic. Trop. Med. Int. Health.

[5] WHO COVID-19 Dashboard. https://covid19.who.int/.

[6] Korber B., Fischer W.M., Gnanakaran S., Yoon H., Theiler J., Abfalterer W., Hengartner N., Giorgi E.E., Bhattacharya T., Foley B. (2020). Tracking changes in SARS-CoV-2 spike: Evidence that D614G increases infectivity of the COVID-19 virus. Cell.

[7] Shrotri M., Swinnen T., Kampmann B., Parker E. (2021). An interactive website tracking COVID-19 vaccine development. Lancet Glob. Health.

[8] Ramasamy M.N., Minassian A.M., Ewer K.J., Flaxman A.L., Folegatti P.M., Owens D.R., Voysey M., Aley P.K., Angus B., Babbage G. (2021). Safety and immunogenicity of ChAdOx1 nCoV-19 vaccine administered in a prime-boost regimen in young and old adults (COV002): A single-blind, randomised, controlled, phase 2/3 trial. Lancet.

[9] Murray C.J., Piot P. (2021). The potential future of the COVID-19 pandemic: Will SARS-CoV-2 become a recurrent seasonal infection?. JAMA.

[10] Nguyen K.H., Srivastav A., Razzaghi H., Williams W., Lindley M.C., Jorgensen C., Abad N., Singleton J.A. (2021). COVID-19 vaccination intent, perceptions, and reasons for not vaccinating among groups prioritized for early vaccination—United States, September and December 2020. Am. J. Transplant..

[11] Sallam M. (2021). COVID-19 Vaccine hesitancy worldwide: A concise systematic review of vaccine acceptance rates. Vaccines.

[12] Kukreti S., Lu M.Y., Lin Y.H., Strong C., Lin C.Y., Ko N.Y., Chen P.L., Ko W.C. (2021). Willingness of Taiwan’s healthcare workers and outpatients to vaccinate against COVID-19 during a period without community outbreaks. Vaccines.

[13] Dror A.A., Eisenbach N., Taiber S., Morozov N.G., Mizrachi M., Zigron A., Srouji S., Sela E. (2020). Vaccine hesitancy: The next challenge in the fight against COVID-19. Eur. J. Epidemiol..

[14] Kwok K.O., Li K.K., Wei W.I., Tang A., Wong S., Lee S.S. (2021). Editor’s choice: Influenza vaccine uptake, COVID-19 vaccination intention and vaccine hesitancy among nurses: A survey. Int. J. Nurs. Stud..

[15] Sherman S.M., Smith L.E., Sim J., Amlôt R., Cutts M., Dasch H., Rubin G.J., Sevdalis N. (2021). COVID-19 vaccination intention in the UK: Rresults from the COVID-19 vaccination acceptability study (CoVAccS), a nationally representative cross-sectional survey. Hum. Vaccin. Immunother..

[16] Biasio L.R., Bonaccorsi G., Lorini C., Pecorelli S. (2021). Assessing COVID-19 vaccine literacy: A preliminary online survey. Hum. Vaccin. Immunother..

[17] daCosta DiBonaventura M., Chapman G.B. (2005). Moderators of the intention–behavior relationship in influenza vaccinations: Intention stability and unforeseen barriers. Psychol. Health.

[18] Rogers R.W. (1975). A Protection motivation theory of fear appeals and attitude change1. J. Psychol..

[19] Maddux J.E., Rogers R.W. (1983). Protection motivation and self-efficacy: A revised theory of fear appeals and attitude change. J. Exp. Soc. Psychol..

[20] Plotnikoff R.C., Trinh L. (2010). Protection motivation theory: Is this a worthwhile theory for physical activity promotion?. Exerc. Sport Sci. Rev..

[21] Bish A., Yardley L., Nicoll A., Michie S. (2011). Factors associated with uptake of vaccination against pandemic influenza: A systematic review. Vaccine.

[22] Cameron K.A. (2009). A practitioner’s guide to persuasion: An overview of 15 selected persuasion theories, models and frameworks. Patient Educ. Couns..

[23] Elhadi M., Msherghi A., Alsoufi A., Buzreg A., Bouhuwaish A., Khaled A., Alhadi A., Alameen H., Biala M., Elgherwi A. (2020). Knowledge, preventive behavior and risk perception regarding COVID-19: Aa self-reported study on college students. Pan. Afr. Med. J..

[24] Al-Hasan A., Khuntia J., Yim D. (2020). Threat, coping, and social distance adherence during COVID-19: Cross-continental comparison sing an online cross-sectional survey. J. Med. Internet Res..

[25] Tan G., Teo I., Anderson K.O., Jensen M.P. (2011). Adaptive versus maladaptive coping and beliefs and their relation to chronic pain adjustment. Clin. J. Pain.

[26] Kok G., Jonkers R., Gelissen R., Meertens R., Schaalma H., de Zwart O. (2010). Behavioural intentions in response to an influenza pandemic. BMC Public Health.

[27] Ezati Rad R., Mohseni S., Kamalzadeh Takhti H., Hassani Azad M., Shahabi N., Aghamolaei T., Norozian F. (2021). Application of the protection motivation theory for predicting COVID-19 preventive behaviors in Hormozgan, Iran: A cross-sectional study. BMC Public Health.

[28] Eppright D.R., Hunt J.B., Tanner J.F., Franke G.R. (2002). Fear, coping, and information: A pilot study on motivating a healthy response. Health Mark. Q..

[29] Rippetoe P.A., Rogers R.W. (1987). Effects of components of protection-motivation theory on adaptive and maladaptive coping with a health threat. J. Pers. Soc. Psychol..

[30] Levin A.T., Hanage W.P., Owusu-Boaitey N., Cochran K.B., Walsh S.P., Meyerowitz-Katz G. (2020). Assessing the age specificity of infection fatality rates for COVID-19: Systematic review, meta-analysis, and public policy implications. Eur. J. Epidemiol..

[31] Bubar K.M., Reinholt K., Kissler S.M., Lipsitch M., Cobey S., Grad Y.H., Larremore D.B. (2020). Model-informed COVID-19 vaccine prioritization strategies by age and serostatus. medRxiv.

[32] Graham B.S. (2020). Rapid COVID-19 vaccine development. Science.

[33] Social Science Statistics. https://www.socscistatistics.com/tests/chisquare2/default2.aspx.

[34] Ministry of Education. https://depart.moe.edu.tw/ed4500/News_Content.aspx?n=5A930C32CC6C3818&sms=91B3AAE8C6388B96&s=B7F6EA80CA2F63EE.

[35] Hu L.T., Bentler P.M. (1999). Cutoff criteria for fit indexes in covariance structure analysis: Conventional criteria versus new alternatives. Struct. Equ. Model..

[36] Rosseel Y. (2012). lavaan: An R package for structural equation modeling and more. Version 0.5–12 (BETA). J. Stat. Softw..

[37] Landau S., Everitt B.S. (2003). A Handbook of Statistical Analyses Using SPSS.

[38] Lin C.Y., Hou W.L., Mamun M.A., Aparecido da Silva J., Broche-Pérez Y., Ullah I., Masuyama A., Wakashima K., Mailliez M., Carre A. (2021). Fear of COVID-19 Scale (FCV-19S) across countries: Measurement invariance issues. Nurs. Open.

[39] Chang K.C., Strong C., Pakpour A.H., Griffiths M.D., Lin C.Y. (2020). Factors related to preventive COVID-19 infection behaviors among people with mental illness. J. Formos. Med. Assoc..

[40] Lin M.W., Cheng Y. (2020). Policy actions to alleviate psychosocial impacts of COVID-19 pandemic: Experiences from Taiwan. Soc. Health Behav..

[41] Bashirian S., Jenabi E., Khazaei S., Barati M., Karimi-Shahanjarini A., Zareian S., Rezapur-Shahkolai F., Moeini B. (2020). Factors associated with preventive behaviours of COVID-19 among hospital staff in Iran in 2020: An application of the Protection Motivation Theory. J. Hosp. Infect..

[42] Li J.B., Yang A., Dou K., Wang L.X., Zhang M.C., Lin X.Q. (2020). Chinese public’s knowledge, perceived severity, and perceived controllability of COVID-19 and their associations with emotional and behavioural reactions, social participation, and precautionary behaviour: A national survey. BMC Public Health.

[43] Teasdale E., Yardley L., Schlotz W., Michie S. (2012). The importance of coping appraisal in behavioural responses to pandemic flu. Br. J. Health Psychol..

[44] Li L., Wang J., Nicholas S., Maitland E., Leng A., Liu R. (2021). The intention to receive the COVID-19 vaccine in China: Insights from protection motivation theory. Vaccines.

[45] Wang P.W., Ahorsu D.K., Lin C.Y., Chen I.H., Yen C.F., Kuo Y.J., Griffiths M.D., Pakpour A.H. (2021). Motivation to have COVID-19 vaccination explained using an extended protection motivation theory among university students in China: The role of information sources. Vaccines.

[46] Pastorino R., Villani L., Mariani M., Ricciardi W., Graffigna G., Boccia S. (2021). Impact of COVID-19 pandemic on flu and COVID-19 vaccination intentions among university students. Vaccines.

[47] Frydenberg E., Lewis R. (2009). Relations among well-being, avoidant coping, and active coping in a large sample of Australian adolescents. Psychol. Rep..

[48] Psychological Responses during COVID-19: Understanding Mental Health, Adaptive and Maladaptive Behaviors. https://www.coronanet-project.org/index.html.

[49] Ling M., Kothe E.J., Mullan B.A. (2019). Predicting intention to receive a seasonal influenza vaccination using protection motivation theory. Soc. Sci. Med..

